# Childhood Mediterranean Diet Compliance Is Associated with Lower Incidence of Childhood Obesity, Specific Sociodemographic, and Lifestyle Factors: A Cross-Sectional Study in Children Aged 6–9 Years

**DOI:** 10.3390/pediatric16040102

**Published:** 2024-12-17

**Authors:** Constantina Jacovides, Agathi Pritsa, Maria Chrysafi, Sousana K. Papadopoulou, Maria G. Kapetanou, Eleftherios Lechouritis, Martin Mato, Vasiliki G. Papadopoulou, Gerasimos Tsourouflis, Athanasios Migdanis, Anastasia Sampani, Rena I. Kosti, Evmorfia Psara, Constantinos Giaginis

**Affiliations:** 1 Department of Food Science and Nutrition, School of the Environment, University of the Aegean, 81400 Lemnos, Greece; con.jacovides@gmail.com (C.J.); m.chrisafi3@gmail.com (M.C.); mariakaptain@yahoo.gr (M.G.K.); lefterislech@gmail.com (E.L.); fns20084@fns.aegean.gr (M.M.); fnsd21013@fns.aegean.gr (E.P.); 2 Department of Nutritional Sciences and Dietetics, School of Health Sciences, International Hellenic University, 57400 Thessaloniki, Greece; agpritsa@ihu.gr (A.P.); souzpapa@gmail.com (S.K.P.); sousana@the.ihu.gr (V.G.P.); 3 Second Department of Propedeutic Surgery, Medical School, University of Athens, 11527 Athens, Greece; gtsourouflis@med.uoa.gr; 4 Department of Nutrition and Dietetics, School of Physical Education, Sport Science and Dietetics, University of Thessaly, 42132 Trikala, Greece; amigdanis@uth.gr (A.M.); renakosti@uth.gr (R.I.K.); 5 First Department of Pathology, Medical School, University of Athens, 11527 Athens, Greece; gerasimos.ts@gmail.com

**Keywords:** childhood obesity, Mediterranean diet, abdominal obesity, anthropometric factors, quality of life, physical activity, breastfeeding, educational status

## Abstract

Background: Mediterranean diet (MD) constitutes a commonly examined dietary model. It includes a plethora of bioactive ingredients with strong antioxidant, anti-inflammatory, antithrombotic and anticancer properties. Several substantial studies support strong evidence that MD can exert preventing actions against human morbidity and mortality, promoting human well-being and quality of life. The present study aims to evaluate whether childhood MD compliance may be associated with socio-demographic, anthropometric, and lifestyle factors in children at the age of 6–9 years. Methods: This is a cross-sectional survey on 3875 children aged 6–9 years old with their matched mothers. Qualified questionnaires were used to evaluate and collect by one-to-one interviews with trained professionals the above data. Results: Elevated MD adherence was observed only in 22.2% of the enrolled children, while 37.5% of children maintained intermediate MD adherence and 40.3% of children adopted lower MD levels. Children MD compliance was related at an independent manner with maternal education level, childhood anthropometric factors such as Body Mass Index (BMI), Waist circumference to Height ratio (WtHR), quality of life, and exclusively breastfeeding behaviors after adjusting for several possible confounders (*p* ˂ 0.05). Conclusions: Elevated MD adherence of children aged 6–9 years old showed a lower obesity of overweight/obesity, including abdominal obesity. A higher maternal educational status and adopting exclusively breastfeeding practices were associated with greater levels of children’s MD adherence, promoting their quality of life and well-being. Based on the present results, future prospective surveys need to be performed to evaluate if there is a causality relation concerning this topic.

## 1. Introduction

The Mediterranean Diet (MD) constitutes a widely recommended and researched dietary pattern for its extensive health benefits. It has a vital role in weight management and the prevention of numerous nutrition-related health diseases such as metabolic syndrome, hypertension, cardiovascular diseases and cancer [1,2]. MD is a dietary model inspired by the traditional eating behaviors of nations bordering the Mediterranean Sea, like Greece, Spain and Italy [1,2]. It is rich in whole grain foods like oats, barley, and whole wheat, fruits, vegetables which are rich in vitamins, minerals, fiber, and antioxidants, legumes, nuts, seeds and olive oil where they provide protein, fiber, and healthy fats [3,4]. The consumption of dairy, fish and poultry is suggested to be in moderate quantities, few times per week coupled with a low consumption of red meat and processed foods to limit saturated fat and sodium intake [5]. MD supports body weight management and decreases the risk of obesity by increasing dietary fiber, promoting satiety, and aiding in blood sugar control, thereby reducing the risk of diabetes type 2 [6]. In addition, several surveys showed that it is linked with a low probability of cardiovascular diseases such as strokes and heart attacks by improving cholesterol levels and reducing inflammation [5,7]. Furthermore, emerging evidence suggests that MD may lower the risk of certain cancers, including colorectal and breast cancer, due to its high antioxidant, fiber, and anti-inflammatory content [8,9].

Childhood overweight and obesity have emerged as critical public health concerns of the twenty-first century worldwide, due to its long-term negative effects [1,4,10]. It has been increasingly commonplace worldwide during the past three to four decades, rising from 4% in 1975 to over 18% in 2016 [11]. Overweight and obesity, is particularly dangerous in Europe, where 29% of children aged 7 to 9 years old met the WHO’s criteria for excess body weight (31% of boys and 28% of girls) in the most recent recommendations of the Childhood Obesity Surveillance Initiative (COSI), which involved 33 countries from Europe [12]. Moreover, people who were overweight as children or adolescents are more likely to experience illness and mortality as adults [13]. Because of this, beyond 2025, the WHO’s member states have vowed to stop future rises in the incidence of childhood obesity [11,12]. The capacity of adipose tissue to deposit fat, which increases in size after too many calories’ consumption, constitutes a highly notable feature of childhood obesity. Adipose tissue volume is influenced by cellular and lipid turnovers, which are closely linked to metabolic health [14]. Nevertheless, it is still unclear how adipose tissue grows and if this affects the equilibrium of the metabolic system [15]. 

There are numerous perinatal, lifestyle, and sociodemographic factors that have been linked with childhood obesity [16,17]. Such factors include family structure, parental education, race/ethnicity, socioeconomic status, and urban or rural living environment [16,17]. Having a thorough understanding of these impacts can aid in creating preventative and intervention plans. According to Shaban Mohamed et al., high maternal body mass index (BMI) before pregnancy and increased childbirth weight are related to a higher risk of developing childhood obesity [18]. In addition, increased gestational weight gain (GWG) might lead to enhanced childbirth weight and fetus macrosomia, which may raise the risk of childhood obesity [19]. Multifaceted factors, including physiological, genetics, environment and behavior of the child, family, and society, contribute significantly to the complex condition of childhood obesity. Regardless of the variety of factors, gestational diabetes mellitus was reported to increase two times the likelihood of overweight/obesity in children aged 2–4 years [20].

Moreover, cultural alterations in nutrition and exercise, along with socioeconomic disparities, contribute to higher rates of obesity [21]. Childhood obesity rates are typically higher in areas with lower socioeconomic status. Families with low incomes may have fewer access to safe places for physical activity and wholesome eating [22,23]. In addition, obesity is more common in children from single-parent households and non-traditional family configurations, such as extended families. These families may experience more financial difficulties and have less time for physical activity and meal preparation [24]. Reduced rates of childhood obesity are typically linked to higher parental education levels. Parents with higher levels of education are more likely to know about and be able to teach their kids healthy living habits [25]. Lastly, compared to children in urban regions, obese children are more likely to live in rural areas. There may be less leisure opportunities, reduced availability of nutritious food, and distinct lifestyles in rural areas [26].

Numerous sociodemographic characteristics, such as parental education, family structure, race/ethnicity, socioeconomic level, and urban against rural living conditions, all have an impact on childhood obesity. It is imperative to address these determinants through customized public health policies and interventions in order to effectively prevent and treat childhood obesity. Childhood adherence to MD has also been found relevant for long-term health, exerting potential role in preventing chronic diseases and obesity in adulthood [6,7,8,9]. However, most of the studies did not focus on a specific age group of children, including mainly a wide age group of children, e.g., 2–12 years old, or including both children and adolescents, e.g., 5–18 years old [6,7,8,9]. In this aspect, this study investigates the association between MD compliance in children aged 6–9 years and socio-demographic, anthropometric, and lifestyle factors, with a focus on maternal influence and breastfeeding practices.

## 2. Materials and Methods

### 2.1. Study Population

**Figure 1:**
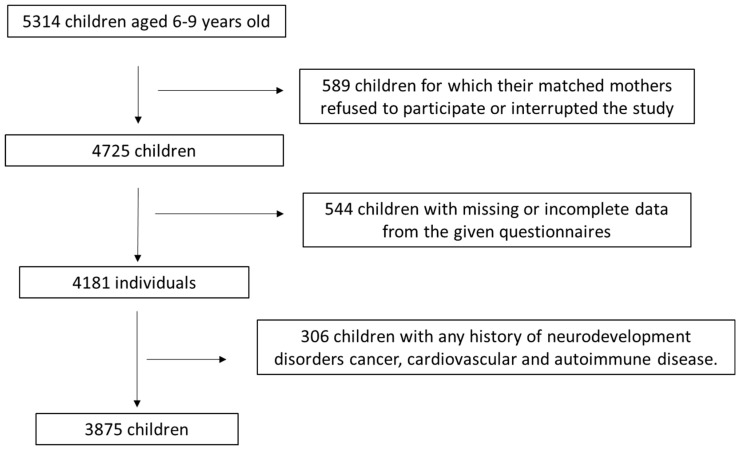
Flow chart diagram for the assigned survey population.

This is a cross-sectional survey that included 5314 children aged 6–9 years old and their matching mothers were primarily assigned from ten geographical areas of Greece, including Athens, Thessaloniki, Larissa, Patra, Alexandroupolis, Kalamata, Ioannina, Crete and South and North Aegean. Enrollment to the survey was randomly performed from May 2018 to September 2021. Most of the mothers were enrolled from primary education schools. The inclusion criteria for the first admission were children at the age of 6–9 years old whose mothers had a singleton childbirth prior to 6–9 years. All enrolled children had not any history of disease except for those diagnosed with childhood asthma or diabetes mellitus I. Amongst the 5314 initially enrolled children and their matched mothers, 589 (11.1%) of their mothers either denied taking part at the survey or they discontinued their involvement to the survey throughout the accomplishment of the undertaken questionnaires. Amongst the continuing 4725 allocated children and their paired mothers, 544 of them (11.5%) were not included in the study because of lost or incomplete data. Amongst the resting 4181 children and their paired mothers, 306 (7.3%) of the enrolled children were not included in the analysis because of any history of childhood neurodevelopment disorders, cancer, cardiovascular and autoimmune diseases. Children disease history was self-reported by their paired mothers in the relevant questionnaires. Subsequently, 3875 children and their matched mothers were ultimately taken part in the survey analysis by utilizing the aforementioned inclusion and exclusion criteria, resulting in a final response rate of 72.9%. A flow chart diagram of the survey assignment is depicted in Figure 1.

All participants’ data remained strictly confidential. The assigned mothers of their paired children were notified concerning the objectives of the study and they engaged a consent form, confirming their approval for potential publishment of their private data anonymously. The study was permitted by the Ethical Agency of the University of the Aegean (ethical approved protocol: no 12/14.5.2016) and was in line with the World Health Organization (52nd WMA General Assembly, Edinburgh, Scotland, 2000). Sample size evaluation was assessed using PS: Power and Sample Size calculator program. The randomization was made applying a sequence of random binary numbers (i.e., 001110110 in which 0 signified assignment and 1 not assignment to the survey). The estimation of the power of the survey sample size showed a power of 88.5%.

### 2.2. Study Design

During the survey, appropriate questionnaires were used for assessing the sociodemographic characteristics of the assigned children like children age, gender (boys vs. girls), nationality (Greek vs. others), and residence kind (urban vs. rural) by face-to-face interviews between their enrolled paired mothers and trained nutritionists or dietitians to decrease recall bias [19,27,28]. The maternal education level was classified into three groups: (a) primary, and (b) secondary education as well as (c) university studies. Financial level was categorized based on the yearly family income as: 0 <5000 €; 1 5000–10,000 €; 2 10,000–15,000 €; 3 15,000–20,000 €; 4 20,000–25,000 €; 5 >25,000 €. Economic level was additionally grouped as low for family yearly income ≤ 10,000 €, medium for yearly income ˃ 10,000 € and ≤20,000 €, and high for yearly income ˃ 20,000 € [19,27,28].

Children anthropometry characteristics like body weight, waist circumference and height were measured during the period of survey by trained staff. Body weight was measured using the same electronic scale, and height was assessed by a portable stadiometer [19,27,28]. The body weight was determined nearly to the closest 100 g, and the height was measured near to the closest 0.50 cm. The International Obesity Task Force (IOTF) recommendations were applied for grouping the enrolled children as normal weight, overweight or obese [29,30]. 

The Waist circumference to Height ratio (WtHR) was also determined by splitting waist with height measurements. Notably, WtHR has been considered as a more effective marker than BMI, reflecting the body fat distribution [31,32]. Especially, it has been considered as an efficient index of abdominal obesity, which has been recognized as an effective anthropometric factor associated more effectually with the probability of developing cardiovascular or metabolic diseases like myocardial infraction, stroke, diabetes mellitus II, etc. [31,32]. WtHR was estimated by dividing WC by height, and a value above 0.50 identified abdominal obesity [31,32]. Childbirth weight was also recovered by mothers’ medical files, being classified as low (<2500 g), normal (2500–4000 g) and high (>4000 g) as suggested by the relevant literature [33].

We also assessed physical activity using the International Physical Activity Questionnaire (IPAQ) in which participants report the duration of any physical activity they do during a representative week. This self-administered questionnaire is utilized worldwide and evaluates the total physical activity concerning the previous seven days, to classify it as low, moderate, or high [34]. IPAQ instruments have thoroughly been examined and showed adequate consistency and validity, at least as good as other self-reported PAQs [34].

The Pediatric Quality of Life Inventory (PedsQL) was used to determine the health-related quality of life of the enrolled children. This is a short questionnaire that is able to effectively be fulfilled by parents [35]. The PedsQL inventory needs about five minutes to be accomplished, while it is able to be self-administered by parents having a child aged ≥2 years old after being thoroughly advised by qualified personnel [35]. PedsQL includes a 23-item generic health state measurement of adequate consistency and validity. In fact, parents, and children forms assess five domains of health (physical functioning, emotional functioning, psychosocial functioning, social functioning, and school functioning) in children and adolescents aged between 2 and 18 years old [35,36]. The PedsQL questionnaire can discriminate amongst healthy children and those suffered from chronic disorders, and it is capable of being related with indices of health care access, days missed from school, days sick in bed or too ill to play, and days needing care [35,36].

Childhood MD adherence of the assigned children was evaluated utilizing the KIDMED questionnaire [37]. The KIDMED questionnaire constitutes one of the most extensively applied scoring systems to evaluate MD compliance. This questionnaire has adequate consistency and validity, including 16 questions for evaluating children’s dietary behaviors. Each question has a “yes” or “no” answer that ranges from −1 (negative connotation) to +1 (positive connotation). Twelve questions take a positive score and four questions take a negative score. Especially, it contains four questions presenting a negative association with the MD (intake of fast food, baked goods, sweets, and skipping breakfast) and twelve questions presenting a positive association with healthful nutritional habits (intake of oil, fish, fruits, vegetables, cereals, nuts, pulses, pasta or rice, dairy products, and yoghurt). An overall KIDMED score ranges from 0 to 12, being categorized as follows: ≥8 points, high; 4–7 points, moderate; and ≤3 points, low MD compliance [37].

Mothers were questioned if they performed exclusive breastfeeding for a minimum duration of four months. To minimize recall bias, the mothers were answered for exclusive breastfeeding for a minimum duration of four months as most of them were recommended to gradually introduce pulp foods to the feeding practices of their children at the end of the 4th month and the beginning of the 5th month and therefore they are capable of remembering more precisely this time point, rendering their answers more reliable. In contrast, mothers breastfeeding for smaller periods, they were not capable of responding with adequate certainty regarding the precise breastfeeding period [38,39]. Childhood asthma was diagnosed by accredited clinicians based on the International Study of Asthma and Allergies in Children and information about asthma-specific therapeutic approach and health care usage [40,41]. Children diagnosed with diabetes mellitus type I data were self-reported by their paired mothers.

Informative comprehensive instructions were methodically specified to the mothers of their assigned children by qualified dietitians and nutritionists regarding the completion of the questionnaires, and an analytical presentation of the questions to achieve consistent answers was utilized for enhancing the validity of mothers’ responses. All questionnaires were responded by the mothers of the enrolled children.

### 2.3. Statistical Analysis

Student’s *t*-test was used concerning the continuous variables, which showed normal distribution. Kolmogorov-Smirnov test was applied for evaluating whether every continuous variable showed a normal distribution. Chi-square was utilized for categorical variables. The normally distributed quantitative variables were expressed by mean value ± Standard Deviation (SD). Non-parametric analysis using Mann-Whitney test was performed for no normally distributed variables. The quantitative no normally distributed continuous variables were expressed by median value (Interquartile Range, IQR). The qualitative variables were expressed as absolute or relative frequencies. To evaluate whether MD may independently be associated with socio-demographic, anthropometric, and lifestyle factors, multivariate binary logistic regression analysis was used after adjustment for potential confounding factors. As confounding factors all socio-demographic, anthropometric, and lifestyle factors were concerned. The Statistica 10.0 software, Europe was applying for the statistical analysis of the data of the present study (Informer Technologies, Inc., Hamburg, Germany).

## 3. Results

### 3.1. Descriptive Statistics of the Study Population

This cross-sectional survey conducted on 3875 children at the age of 6–9 years old. All the descriptive statistics were included in Table 1. The mean age of the enrolled children was 7.4 ± 1.6 years old. 49.8% of the children were boys and 50.2% were girls. The vast majority (95.5%) of the enrolled children had Greek nationality, while 4.5% of them had other nationality (Albanian, Russian, Bulgarian, Arabian and other nationalities). 66.6% of the participating children lived in urban areas in Greece and the remaining 33.4% lived in rural areas. 29.8% of the paired mothers had finalized primary education studies and 42.9% had received secondary education studies. A proportion of 27.3% of the enrolled mothers had finalized university studies. 44.1% of the matched mothers reported a low financial level, 42.0% of them stated a medium financial level, and only 13.9% of the participating mothers had a high financial level.

As far as the anthropometry characteristics as concern, according to BMI, 16.7% of the enrolled children were overweight and 7.2% were obese, and therefore 23.9% were overweight/obese overall. According to WtHR, 26.5% of the enrolled children had abdominal obesity and the remaining 73.5% had not. Regarding childbirth weight, 8.0% of the participating children exhibited high birth weight, and another 8.0% exhibited low birth weight. 

Regarding physical activity levels evaluated by Pre-PAQ, 47.8% of the children showed low and 39.9% showed medium physical activity levels, while merely 12.3% of the assigned children had high physical activity levels. The mean quality of life score evaluated by PedsQL questionnaire was 63.7 ± 7.2. In addition, 7.5% of the participating children were diagnosed with asthma and 6.9% of them suffered from diabetes mellitus type 1. 50.1% of the participating children breastfed exclusively for a minimum period of four months. According to KIDMED questionnaire, merely 22.2% of the enrolled children presented high levels of MD compliance, while 37.5% of the children exhibited moderate MD aherence and 40.3% of children presented low MD compliance.

### 3.2. Association of Children MD Adherence with Sociodemographic Characteristics

Boys showed considerably greater levels of MD compliance than girls (Table 2, *p* = 0.0358), whereas MD compliance of children was not related with children age (Table 2, *p* ˃ 0.05). Greek children presented greater MD adherence than children of other nationalities (Table 2, *p* = 0.0057). Children living in rural areas had considerably elevated levels of MD compliance than children living in urban areas (Table 2, *p* ˂ 0.0001). Mothers’ education level was positively related to MD compliance (*p* = 0.0012). Moreover, a better family economic status was significantly related with higher levels of MD adherence (*p* = 0.0010). 

### 3.3. Association of Children MD Adherence with Anthropometric Characteristics

Children which were classified as overweight, or obese, exhibited considerably decreased levels of MD adherence compared to normal weight children (Table 2, *p* ˂ 0.0001). Abdominal obesity classified by WtHR was significantly more frequently observed in children with lower levels of MD compliance compared to those without abdominal obesity (Table 2, *p* ˂ 0.0001). 

### 3.4. Association of Children MD Compliance with Lifestyle Factors

Greater MD compliance was considerably more frequently noted in children with higher physical activity levels and better quality of life scores (Table 2, *p* = 0.0006 and *p* = 0.0012, respectively). Exclusive breastfeeding was substantially more frequently observed in children presenting greater MD adherence than those who did not breastfeed at all or breastfed lower than four months (Table 2, *p* ˂ 0.0001). 

### 3.5. Association of Children MD Compliance with Childhood Asthma and Diabetes Mellitus 1

Children suffered from asthma or diabetes mellitus type 1 presented substantially poorer MD compliance compared to children without asthma or diabetes mellitus type 1 (Table 2, *p* = 0.0004, and *p* = 0.0001, respectively)

### 3.6. Multivariate Binary Logistic Regression Analysis for MD Compliance of the Study Population

In multivariate binary logistic regression analysis, children MD compliance was independently related with maternal education level, anthropometry indices such as BMI, and WtHR as well as with quality of life, and breastfeeding practices (*p* ˂ 0.05). Specifically, children presenting low or moderate MD adherence presented a more than a 2-fold higher incidence of overweight or obesity than children with high MD compliance (Table 3, OR: 2.18, CI: 1.91–2.46, *p* = 0.0071). Children with low or moderate MD adherence exhibited more than a two-fold greater prevalence of abdominal obesity compared to those presenting elevated MD compliance (Table 3, OR: 2.07, CI: 1.75–2.44, *p* = 0.0179).

Children whose paired mothers had a greater educational level had a 48% greater incidence of adopting enhanced levels of MD adherence (Table 3, OR: 1.48, CI: 1.17–1.72, *p* = 0.0204). Children with low or moderate MD adherence exhibited a more than two-fold prevalence of lower rates of quality of life than those presenting elevated MD adherence (Table 3, OR: 2.12, CI: 1.81–2.38, *p* = 0.0105). Children exclusively breastfeeding for a minimum period of four months had a more than two-fold prevalence of higher MD compliance than those with low or moderate MD adherence (Table 3, OR: 2.17, CI: 1.92–2.39, *p* = 0.0081). 

In contrast, children age, gender, nationality, type of residence, family financial status, birth weight, physical activity, childhood asthma and diabetes mellitus type 1 were not independently related with MD compliance (Table 3, *p* > 0.05).

## 4. Discussion

The present study aimed to evaluate the association of childhood MD adherence with socio-demographic, anthropometric, and lifestyle factors in Greek children aged 6–9 years. Our findings indicated that higher adherence to MD was considerably correlated with lower BMI and waist circumference, suggesting a protective effect against obesity. The study also found significant associations between maternal education, quality of life and breastfeeding practices with both MD adherence and childhood obesity rates. Specifically, children from families with higher maternal education and children who breastfed exclusively for at least 4 months were more likely to adhere to the MD and less likely to be affected by obesity. In addition, children who presented higher rates of quality of life were considerably more likely to adhere to the MD.

Children with asthma or diabetes type 1 had notably lower adherence to MD than their peers without these conditions. Boys demonstrated substantially greater levels of MD adherence than girls, while Greek children exhibited greater MD compliance than children of other nationalities. Children engaged in more physical activity, residing in rural areas, and with better family economic status showed higher MD adherence compared to those living in urban regions. However, after adjusting for various confounding factors, these associations were significantly weakened and became non-significant. Moreover, it should be noted that only 22.2% of the assigned children had higher rates of elevated MD adherence. This finding means that most children may have an increased risk of developing any chronic disease at the next stages of their life. Although we did not find a significant association in multivariate analysis, the socioeconomic level of mothers has also been associated with the dietary habits of both the mothers and their matched children in several studies [42,43,44].

Aligning with the results by a prior study on Greek children and adolescents, our sample exhibited relatively low adherence to the MD. Nonetheless, in both studies, children who adhered to the MD showed a lower prevalence of overweight and obesity [42]. A cross-sectional study among pre-school children living in different regions of Greece, showed that higher adherence to the MD was independently correlated with a reduced prevalence of both childhood overweight and obesity, as well as a lower incidence of abdominal obesity [43]. Additionally, greater MD compliance was independently associated with increased physical activity levels, enhanced quality of life, exclusive breastfeeding, and a decreased prevalence of childhood asthma and diabetes mellitus type 1 in pre-school children [43]. Moreover, the results from the IDEFICS study, one of Europe’s largest children’s cohorts, indicated that children residing in Mediterranean countries have increasingly departed from this traditional dietary pattern, which was inversely correlated with childhood overweight and obesity [44]. 

As demonstrated by a nationwide study, childhood obesity prevalence in Greece reaches record highs, accompanied by notably low adherence rates to the MD. This study reported that only 4.3% of the children attained an optimal KIDMED score. Children with higher KIDMED scores reported healthier eating habits and higher levels of physical activity [45]. In our study, a higher percentage of 22.2% of the assigned children had higher KIDMED score; however, this percentage remain low concerning that MD is a traditional diet for Mediterranean countries, including our country Greece. In line with our findings in a prior study that included students from Greek regions, showed that overweight and obese children had lower KIDMED scores compared to normal-weight children. An inverse association between KIDMED scores and weight status was observed, particularly in nuclear families, whereas this relationship was not significant in single-parent families [46]. Moreover, the same study demonstrated that children’s adherence to the MD was negatively associated with their body weight status, irrespective of their parents’ adherence to the diet [47]. Additionally, a recent study by Zheng et al., that used data from the National Health and Nutrition Examination Survey (NHANES) supported evidence that greater MedDiet scores were linked to a decreased likelihood of overweight and obesity in children and adolescents [48].

The ΕΥΖHΝ study, which included 174,209 students from all geographical regions of Greece, used the MediLIFE-index as an indicator of the Mediterranean lifestyle, encompassing MD adherence, levels of physical activity, sedentary behavior, and sleep duration concurrently [49]. Students with higher MediLIFE-index scores exhibited considerably lower BMI and waist circumference. Specifically, those classified as “highly adherent” to the MediLIFE-index were associated with a 6% lower likelihood of being overweight, obese, or abdominally obese compared to those categorized as “non-adherent” [49]. The results of SENDO project, a pediatric cohort study in Spain, showed that after adjusting for various sociodemographic and lifestyle factors, such as parental attitudes and knowledge regarding children’s dietary guidelines, breastfeeding emerged as independently associated with greater adherence to the MD [50]. Specifically, children breastfed for six months or more exhibited an average increase of one point in their KIDMED score than those who were never breastfed [50]. In accordance with our study, mothers with higher education who applied breastfeeding practices are more likely to adopt a healthier diet for both their children and themselves.

The results of a recent systematic review supported substantial evidence for a protective effect of the MD against childhood asthma, while indicating no significant impact on the development of allergies. However, the researchers highlighted that the heterogeneity and limitations across the existing studies underscore the necessity for designing and performing randomized controlled trials focusing on pediatric populations to yield more robust evidence [51]. The findings from an umbrella review highlight that adherence to the MD is associated with a protective effect against childhood asthma, likely attributed to its antioxidant-rich components [4]. Moreover, in agreement with our results, it was found a positive correlation between MD adherence and enhanced physical activity levels, improved physical fitness, better quality of life, and reduced sedentary behavior among children [4]. Additionally, MD seems to exhibit an inverse relationship with pro-inflammatory biomarkers, indicating potential anti-inflammatory properties [52].

There is robust evidence that adherence to MD is associated with lower obesity rates The MD’s emphasis on whole, minimally processed foods, rich in fiber, vitamins, minerals, and healthy fats, likely contributes to these benefits [53]. MD is universally recognized as an ideal dietary pattern for preventing chronic diseases and promoting overall health [54]. Its focus on plant-based foods such as fruits, vegetables, whole grains, legumes, and nuts provides a rich source of essential nutrients and antioxidants that can reduce inflammation and improve metabolic health [54,55]. The inclusion of healthy fats, primarily from olive oil, its rich supply of omega-3 fatty acids, and the moderate consumption of dairy products and lean proteins like fish, may further promote cardiovascular health and cognitive function and development [56,57]. Additionally, the MD’s balanced approach to nutrition helps establish healthy eating habits that can last a lifetime, promoting long-term well-being.

Collectively, MD is not only effective in reducing obesity rates among children but also offers a comprehensive approach to nutrition which can support overall health and development. The findings underscore the importance of promoting MD among children to combat obesity and improve overall health. Public health initiatives should focus on educating parents, especially those with lower educational and economic status, about the benefits of the MD. Its emphasis on whole, nutrient-dense foods and healthy fats, combined with its cultural and social dimensions, make it an ideal dietary pattern for promoting a healthy lifestyle from childhood through adulthood.

A major strength of this study is the large sample size from diverse regions across our country, which enhances the generalizability of our findings. We conducted comprehensive evaluations encompassing various sociodemographic, anthropometric, and lifestyle factors, underscoring the depth of our research approach. To minimize recall bias, we conducted one-on-one interviews with trained staff and mothers of participating children, ensuring thorough explanations and detailed questionnaire presentations. Anthropometric measurements were meticulously performed by qualified personnel, ensuring the accuracy and reliability of the data over self-reported information. Validated questionnaires were utilized to assess MD adherence, quality of life, and physical activity levels among the children, complemented by childhood asthma diagnoses confirmed by specialized physicians. Importantly, our study expanded beyond basic BMI classification to include WC and WtHR assessments, providing a more comprehensive evaluation of abdominal obesity. These measures are pivotal as they offer predictive insights into the risk of chronic diseases in later life stages. Together, these methodological strengths underscore the credibility and applicability of our findings.

However, there are limitations to consider when interpreting our findings. The cross-sectional nature of our study design restricts our ability to establish definitive causal relationships between MD adherence and childhood overweight/obesity. Thus, we must emphasize that the cross-sectional design limits causal inferences. Despite our efforts to minimize recall bias through rigorous one-on-one interviews, the reliance on self-reported data introduces potential confounding factors, although such data are commonly used and generally reliable in epidemiological studies. Hence, we must emphasize that the self-reported data from questionnaires may lead to potential biases. Moreover, our adjustment for confounders may not encompass all relevant variables, such as aspects of mental health, sleep patterns, and the presence of eating disorders among children, which could influence our results as residual confounders. The questionnaires used in our study were completed by mothers, who may have provided biased responses reflecting their own perceptions of their children’s dietary habits. Addressing these limitations in future studies will be essential for advancing our understanding of how MD adherence impacts childhood health outcomes, including obesity prevention strategies.

Future research should prioritize longitudinal studies to track MD adherence from early childhood through adolescence and into adulthood. These studies would offer valuable insights into how sustained adherence to the MD may influence long-term weight management and metabolic health outcomes. Additionally, interventions promoting the MD in school settings should be rigorously evaluated for their effectiveness in reducing childhood obesity rates. Exploring the biological mechanisms underlying the protective effects of MD, such as its impact on gut microbiota composition and metabolic pathways, could provide deeper insights into its potential role in obesity prevention.

Furthermore, examining the interaction between cultural and socio-demographic factors and MD adherence could identify vulnerable populations who may benefit most from targeted dietary interventions. Understanding how factors such as socioeconomic status, parental education, and family structure can influence dietary habits and obesity risk is crucial for developing tailored public health strategies. The role of psychosocial factors, including parental feeding practices, family meal environments, and peer influences, on children’s adherence to the MD and subsequent obesity outcomes should be investigated. Additionally, expanding research to diverse populations beyond Greece will help generalize the findings and inform global dietary recommendations. Identifying barriers to MD adherence and developing strategies to overcome these challenges can enhance the effectiveness of public health initiatives aimed at promoting MD and reducing childhood obesity rates.

## 5. Conclusions

In conclusion, this study highlights the beneficial role of MD in preventing childhood obesity and highlights the importance of promoting this dietary pattern among young children. By addressing sociodemographic and lifestyle factors, comprehensive approaches can be developed to combat childhood obesity and promote long-term health. Further research is essential to explore the enduring benefits of MD adherence, elucidate the underlying biological mechanisms, and identify effective strategies for promoting this dietary pattern across diverse populations. These findings advocate for public health strategies that encourage adherence to the MD from an early age, potentially laying the foundation for healthier future generations. The findings of the present study may also activate public health initiatives, such as the potential for targeted nutrition education programs for families. Moreover, future longitudinal studies should explore causal relationships and examine the long-term effects of MD adherence on childhood obesity and quality of life.

## Figures and Tables

**Table 1:** Descriptive statistics of the enrolled children aged 6–9 years old.

Characteristics (n = 3875)	Descriptive Statistics
**Age (mean ± SD; years)**	7.4 ± 1.6
**Gender (n, %)**	
Male	1930 (49.8%)
Female	1945 (50.2%)
**Nationality (n, %)**	
Greek	3701 (95.5%)
Other	174 (4.5%)
**Type of residence (n, %)**	
Urban	2581 (66.6%)
Rural	1294 (33.4%)
**Maternal educational level**	
Primary education	1155 (29.8%)
Secondary education	1664 (42.9%)
University studies	1056 (27.3%)
**Family economic status**	
Low	1708 (44.1%)
Medium	168 (42.0%)
High	539 (13.9%)
**BMI (n, %)**	
Normal weight	2924 (75.5%)
Overweight	647 (16.7%)
Obese	304 (7.8%)
**Abdominal obesity status (WtHR) (n, %)**	
No	2849 (73.5%)
Yes	1026 (26.5%)
**Birth weight (n, %)**	
Low weight (<2500 g)	309 (8.0%)
Normal weight (2500–4000 g)	3257 (84.0%)
High weight (>4000 g)	309 (8.0%)
**Preterm birth**	
No	3037 (78.4%)
Yes	838 (21.6%)
**Physical activity ^1^ (n, %)**	
Low	1852 (47.8%)
Medium	1547 (39.9%)
High	478 (12.3%)
**Quality of life ^2^ (mean score ±SD)**	63.7 ± 7.2
**Exclusive breastfeeding (n, %)**	
No	1942 (50.1%)
Yes	1933 (49.9%)
**Asthma (n, %)**	
No	3584 (92.5%)
Yes	291 (7.5%)
**Diabetes type 1 (n, %)**	
No	3606 (93.1%)
Yes	269 (6.9%)
**KIDMED ^3^ score (n, %)**	
Low	1562 (40.3%)
Moderate	1453 (37.5%)
High	860 (22.2%)

^1^ Physical activity levels were evaluated by the International Physical Activity Questionnaire (IPAQ) [34]. ^2^ Quality of life was evaluated by the Pediatric Quality of Life Inventory (PedsQL) questionnaire [35,36]. ^3^ Mediterranean diet (MD) compliance was determined by the KIDMED (Mediterranean Diet Quality Index for children and adolescents) questionnaire [37].

**Table 2:** Associations of Mediterranean diet adherence (KIDMED) with socio-demographic, anthropometric and lifestyle factors of the assigned children aged 6–9 years old.

Characteristics (n = 3875)	KIDMED ^3^ Score			
	Low 1562 (40.3%)	Moderate 1453 (37.5%)	High 860 (22.2%)	*p*-Value
**Age (mean ± SD; years)**	7.38 ± 1.11	7.41 ± 1.08	7.40 ± 1.09	*p* = 0.2576
**Gender (n, %)**				*p* = 0.0358
Boys	745 (47.7%)	728 (50.1%)	457 (53.1%)	
Girls	817 (52.3%)	725 (49.9%)	403 (46.9%)	
**Nationality (n, %)**				*p* = 0.0057
Greek	1472 (94.2%)	1397 (96.1%)	832 (96.7%)	
Other	90 (5.8%)	56 (3.9%)	28 (3.3%)	
**Type of residence (n, %)**				*p* ˂ 0.0001
Urban	1082 (69.3%)	1086 (74.7%)	413 (48.0%)	
Rural	480 (30.7%)	367 (25.3%)	447 (52.0%)	
**Maternal educational level**				*p* = 0.0012
Primary education	509 (32.6%)	392 (27.0%)	254 (29.5%)	
Secondary education	667 (42.7%)	652 (44.9%)	345 (40.1%)	
University studies	386 (24.7%)	409 (28.1%)	261 (30.3%)	
**Family economic status**				*p* = 0.0010
Low	691 (44.2%)	652 (44.9%)	365 (42.5%)	
Medium	685 (43.9%)	603 (41.5%)	340 (39.5%)	
High	186 (11.9%)	198 (13.6%)	155 (18.0%)	
**BMI (n, %)**				*p* ˂ 0.0001
Normal weight	842 (53.9%)	1288 (88.7%)	794 (92.3%)	
Overweight	492 (31.5%)	105 (7.2%)	50 (5.8%)	
Obese	228 (14.6%)	60 (4.1%)	16 (1.9%)	
**Abdominal obesity (WtHR) (n, %)**				*p* ˂ 0.0001
No	803 (51.4%)	1282 (88.2%)	764 (88.8%)	
Yes	759 (48.6%)	167 (11.8%)	96 (11.2%)	
**Birth weight (n, %)**				*p* = 0.0011
Low birth weight (<2500 g)	109 (7.0%)	115 (7.9%)	83 (9.5%)	
Normal birth weight (2500–4000 g)	1292 (82.7%)	1232 (84.8%)	730 (84.9%)	
High birth weight (>4000 g)	161 (10.3%)	106 (7.3%)	47 (5.6%)	
**Physical activity ^1^ (n, %)**				*p* = 0.0006
Low	784 (50.2%)	701 (48.2%)	367 (42.7%)	
Moderate	612 (39.2%)	578 (39.8%)	357 (41.5%)	
High	166 (10.6%)	174 (12.0%)	136 (15.8%)	
**Quality of life ^2^ (mean score ± SD)**	61.5 ± 6.7	63.2 ± 7.1	66.5 ± 7.6	*p* = 0.0012
**Exclusive breastfeeding (n, %)**				*p* ˂ 0.0001
No	868 (55.6%)	689 (47.4%)	385 (44.8%)	
Yes	694 (44.4%)	764 (52.6%)	475 (55.2%)	
**Asthma (n, %)**				*p* = 0.0004
No	1411 (90.3%)	1355 (93.3%)	818 (95.1%)	
Yes	151 (9.7%)	98 (6.7%)	42 (4.9%)	
**Diabetes type 1 (n, %)**				*p* = 0.0001
No	1410 (90.3%)	1373 (94.5%)	823 (95.7%)	
Yes	152 (9.7%)	80 (5.5%)	37 (4.3%)	

^1^ Physical activity levels were evaluated by the International Physical Activity Questionnaire (IPAQ) [34]. ^2^ Quality of life was evaluated by the Pediatric Quality of Life Inventory (PedsQL) questionnaire [35,36]. ^3^ Mediterranean diet (MD) compliance was determined by the KIDMED (Mediterranean Diet Quality Index for children and adolescents) questionnaire [37].

**Table 3:** Multivariate logistic regression analysis for MD adherence of children aged 6–9 years old.

Characteristics	Mediterranean Diet ^1^ Adherence (Low vs. Moderate & High)	
	OR * (95% CI **)	*p*-Value
**Age** (Over/Below mean value)	0.98 (0.23–1.74)	*p* = 0.5748
**Gender** (Boys/Girls)	1.21 (0.60–1.99)	*p* = 0.2018
**Nationality** (Greek/Other)	1.16 (0.54–1.99)	*p* = 0.3103
**Type of residence** (Rural/Urban)	1.07 (0.46–1.75)	*p* = 0.4019
**Maternal educational level** (University studies/Primary & Secondary education)	1.48 (1.17–1.72)	*p* = 0.0204
**Family economic status** (High/Low & Medium)	1.31 (0.82–1.89)	*p* = 0.1049
**BMI** (Overweight & Obese/Normal weight)	2.18 (1.91–2.46)	*p* = 0.0071
**Abdominal obesity** (WtHR) (No/Yes)	2.07 (1.75–2.44)	*p* = 0.0179
**Birth weight** (Low & normal/High)	1.69 (0.91–2.48)	*p* = 0.2906
**Physical activity ^2^** (Low/Moderate & High)	1.62 (1.03–2.11)	*p* = 0.1547
**Quality of life ^3^** (Below/Over mean value)	2.12 (1.81–2.38)	*p* = 0.0105
**Exclusive breastfeeding** (No/Yes)	2.17 (1.92–2.39)	*p* = 0.0081
**Asthma** (No/Yes)	1.51 (0.95–2.14)	*p* = 0.2209
**Diabetes type 1** (No/Yes)	1.34 (0.59–2.2)	*p* = 0.3762

* Odds Ratio: OR ** CI: Confidence Interval ^1^ Mediterranean diet (MD) compliance was determined by the KIDMED (Mediterranean Diet Quality Index for children and adolescents) questionnaire [37]. ^2^ Physical activity levels were assessed by the International Physical Activity Questionnaire (IPAQ) [34]. ^3^ Quality of life was evaluated by the Pediatric Quality of Life Inventory (PedsQL) questionnaire [35,36].

## Data Availability

The data of the study is available upon request to the corresponding author.
